# Bridging the Digital Gap for Older Adults in Korea: Direct Care Workers as Mediators

**DOI:** 10.1093/geroni/igaf122.2795

**Published:** 2025-12-31

**Authors:** Jeongone Seo, Kyung-Zoon Hong, Sol Baik

**Affiliations:** Sungkyukwan University, Seoul, Republic of Korea; Sungkyukwan University, Seoul, Republic of Korea; University of Virginia, Charlottesville, Virginia, United States

## Abstract

In the face of rapid aging in South Korea, information and communication technologies (ICTs) hold significant potential for improving older adults’ well-being and social engagement. Yet many older adults experience barriers such as low digital literacy, device anxiety, and fear of scams. This study explored direct care workers’ experiences facilitating digital usage of older adults in the community. We conducted semi-structured interviews with ten direct care workers at adult daycare and home care settings in Korea. Our qualitative thematic analysis revealed that care workers played pivotal roles as demonstrators, troubleshooters, and motivators. Many older adults, especially those with mild cognitive impairments, relied on hands-on demonstrations and repeated, step-by-step guidance with emotional support from care workers. While care workers viewed tools (e.g., smartphones, tablets, and AI-based devices) as helpful in enhancing the social connections of older adults and monitoring their safety, they also expressed concerns about privacy, data security, and increased workloads. Furthermore, financial constraints and limited formal training hindered care workers’ effectiveness. Findings suggest that older adults benefit most when digital solutions are tailored to their familiar interests, combined with person-centered teaching strategies. For care workers, targeted training and stronger institutional support are crucial to fostering technology adoption. By emphasizing the direct care workers’ mediator role, this study contributes practical insights for better integrating digital health and social tools into long-term care settings in Korea. Future research will expand the findings with larger samples and cross-cultural comparisons to refine strategies for empowering older adults with effective digital usage.

